# Exploring the cognitive development of children born to adolescent mothers in South Africa

**DOI:** 10.1002/icd.2408

**Published:** 2023-03-07

**Authors:** Kathryn J. Steventon Roberts, Colette Smith, Elona Toska, Lucie Cluver, Camille Wittesaele, Nontokozo Langwenya, Yulia Shenderovich, Wylene Saal, Janina Jochim, Jenny Chen‐Charles, Marguerite Marlow, Lorraine Sherr

**Affiliations:** ^1^ Department of Social Policy and Intervention University of Oxford UK; ^2^ Institute for Global Health University College London UK; ^3^ Centre for Social Science Research University of Cape Town South Africa; ^4^ Department of Sociology University of Cape Town South Africa; ^5^ Department of Psychiatry and Mental Health University of Cape Town South Africa; ^6^ London School of Hygiene and Tropical Medicine UK; ^7^ Wolfson Centre for Young People's Mental Health Cardiff University UK; ^8^ Centre for the Development and Evaluation of Complex Interventions for Public Health Improvement (DECIPHer), School of Social Sciences Cardiff University UK; ^9^ School of Humanities Sol Plaatje University South Africa; ^10^ Institute of Life Course Health Research Stellenbosch University South Africa

**Keywords:** adolescent motherhood, child cognitive development, South Africa, sub‐Saharan Africa

## Abstract

This study explores the cognitive development of children born to adolescent mothers within South Africa compared to existing reference data, and explores development by child age bands to examine relative levels of development. Cross‐sectional analyses present data from 954 adolescents (10–19 years) and their first‐born children (0–68 months). All adolescents completed questionnaires relating to themselves and their children, and standardized child cognitive assessments (Mullen Scales of Early Learning) were undertaken. Cognitive development scores of the sample were lower than USA reference population scores and relative performance compared to the reference population was found to decline with increasing child age. When compared to children born to adult mothers in the sub‐Saharan African region, children born to adolescent mothers (human immunodeficiency virus [HIV] unexposed; *n* = 724) were found to have lower cognitive development scores. Findings identify critical periods of development where intervention may be required to bolster outcomes for children born to adolescent mothers.

**Highlights:**

- An exploration of the cognitive development of children born to adolescent mothers within South Africa utilizing the Mullen Scales of Early Learning.
- Cognitive development scores of children born to adolescent mothers within South Africa were lower compared to USA norm reference data and declined with child age.
- Previous studies utilizing the Mullen Scales of Early Learning within sub‐Saharan Africa were summarized, and comparisons were made with the current sample.
- Findings highlight a potential risk of developmental delay among children born to adolescent mothers compared to children of adult mothers in the sub‐Saharan African region.

## 1 INTRODUCTION

Adolescent mothers (10–19 years) account for ~10% of births globally (Mayor, 2004; Organization, 2021). Adolescent motherhood remains a prominent health and social care issue that demands attention to ensure the success and prosperity of both adolescents and their children (Groves et al., 2018; Toska et al., 2020). Avoiding poor child development, particularly within low‐ and middle‐income countries, remains a core priority of the Sustainable Development Goals and, as such, a core priority for global health research. Within sub‐Saharan Africa, children are often exposed to multiple forms of deprivation (Bain et al., 2013; Goldberg & Short, 2016; Hotez & Kamath, 2009; Mudogo, 2017). As such, over two thirds of children (approximately 66%) under 5 years of age do not reach their cognitive potential, making children living in sub‐Saharan Africa at the highest risk of poor child development globally (Lu et al., 2016).

The attainment of skills and prospects throughout the life course is constructed on foundational capacities formed in early childhood (Daelmans et al., 2017). Child cognition has lasting implications for both the individual child themselves as they move through childhood, adolescence, and adulthood, and additionally has implications for broader communities and societies. Not reaching developmental potential is potentially associated with poor economic implications (e.g., children who do not reach their developmental potential are anticipated to receive only three quarters of the average annual income in adulthood compared to their peers who did reach their developmental potential; Daelmans et al., 2017). Consequently, developmental delay may preserve cycles of poverty within future generations, which in turn may have widespread effects on regional and national growth, gross domestic product, and country contributions to the global economy (Daelmans et al., 2017; Richter et al., 2017). Therefore, promoting the successful development of children within the sub‐Saharan African region is essential to ensuring the enduring success of the individual, the region, and more broadly, the global economy. It therefore remains critical to identify needs, and those groups that may be particularly vulnerable.

Children born to adolescent mothers have been found to be at an increased risk of adversity (Barlow et al., 2011), including poorer neonatal, cognitive, behavioural, and educational outcomes (Branson et al., 2015; Jutte et al., 2010). There is a rapid period of growth within early childhood. Child cognitive development is impacted by numerous factors inclusive of but not limited to biological factors (e.g., birth weight, nutrition, health conditions—such as human immunodeficiency virus [HIV] [Rochat et al., 2017]), environmental factors (e.g., home environment, socio‐economic resources [income, educational attainment]), and psychosocial factors (e.g., parental health, parent–child interaction, learning and development opportunities [Engle et al., 2011; Grantham‐McGregor et al., 2007; Walker et al., 2011]). Hence, child cognition is particularly vulnerable during early development, and it is during this period that interventions to bolster outcomes and limit the impact of adversity may be most effective (Doyle et al., 2007; Doyle et al., 2009; Grantham‐McGregor et al., 2007; Jeong et al., 2021). However, there are differing rates of neural maturation during this early period, with different skills and abilities (i.e., motor/perception skills) becoming prominent at different ages (Milosavljevic et al., 2019). As such, global measures of development may only represent a partial picture of child cognitive development. Tracking age‐related differences in development (inclusive of differing skills and abilities [e.g., motor, perception, language]) may be important in identifying key periods of development that may be sensitive to adversity and, likewise, sensitive to interventions aimed at bolstering development (Milosavljevic et al., 2019).

Adolescent pregnancy is defined by maternal age, but may signify the combination of multiple other adversities, clustering around poverty, education, violence, HIV, and risk behaviours. There is a dearth of literature exploring the cognitive development of the children born to these adolescent mothers within sub‐Saharan Africa. There is little fine‐grained insight into various needs and possible drivers, resulting in a poor understanding of the range of possible needs within such populations, with the resultant lack of evidence‐based policy and programming for this group. Generalizing existing interventions for different populations may provide useful strategies. Comparing cognitive performance with existing reference data may be useful in assessing where need may exist.

This study aims to explore the cognitive development of children born to adolescent mothers in South Africa (measured using the Mullen Scales of Early Learning; Mullen, 1995) compared to a normative reference populations, both from validated normative standards from children residing in the United States (USA) and from additional analyses compared with the cognitive development scores of children born to adult mothers in the sub‐Saharan African region (pooled and weighted data drawn from a systematic search of studies utilizing the Mullen Scales of Early Learning [Mullen, 1995] in the sub‐Saharan African region). The study then aims to further understand the child development trajectory by making comparisons according to child age (utilizing age bands) to identify key periods of development for children of adolescent mothers (0–68 months).

## 2 METHODS

### 2.1 Participants and procedure

Data utilized within these analyses was drawn from a large cohort study of adolescent and young mothers (10–24 years of age) and their child(ren) residing in rural and peri‐urban health districts of the Eastern Cape province, South Africa (*n* = 1046 mothers; the *Helping Empower Youth Brought up in Adversity with their Babies and Young children* [HEY BABY] study). Mothers who had their first child before the age of 20, with at least one living child, were interviewed between March 2018 and July 2019. Six parallel sampling strategies developed with an advisory group of adolescent mothers and local experts (including health facilities [*n* = 73], secondary schools [*n* = 43], service provider referrals, maternity obstetric units [*n* = 9], neighbouring adolescents of participants, and referrals from adolescent mothers) were utilized to maximize recruitment.

All mothers (and their caregivers, if participants were < 18 years of age) provided informed, voluntary consent. Additional consent was obtained from the child's primary caregiver if the adolescent/young mother identified that they were not the main caregiver for their child. All data collection tools were piloted with adolescent mothers (*n* = 9) and adolescents living with HIV (*n* = 25). Three components of data were collected: (1) A maternal detailed study questionnaire consisting of validated scales and study specific questions inclusive of sociodemographic characteristics, health (inclusive of mental health screening measures), relationships, community, and, management of HIV (if applicable), (2) Participants completed a caregiver questionnaire collecting data on health (both maternal and child), access to care, support, education, child development, parenthood experience, and the father of their child(ren) and, (3) Standardized cognitive assessments of children were completed using the Mullen Scales of Early Learning. All questionnaires were administered using electronic tablets facilitated by trained data collectors. Participants completed all components of data collection in their language of choice (isiXhosa or English) and data as translated and back translated as appropriate.

Ethical approvals were obtained from the Universities of Cape Town (HREC 226/2017), Oxford (R48876/RE002), and University College London (14,795/001). Additional local approvals and permissions were obtained from partaking education and health facilities as well as the Provincial Departments (Eastern Cape, South Africa) of Health, Education, and Social Development.

These analyses only present data for adolescent mothers (mothers who had given birth between the ages of 10–19 years) and their first‐born children (≤68 months; in keeping with the validated age range of the Mullen Scales of Early Learning [Mullen, 1995]). Young mothers who gave birth outside of the adolescent age range of 10–19 years, children above 68 months of age at the time of the questionnaire, and second/third‐born children were excluded from analyses. Given the high prevalence of HIV in the sample (24.1%) and the known impacts of living with HIV on child cognitive development (Sherr et al., 2009), those children who were identified as living with HIV or their HIV status was unknown, were also excluded from analyses (*n* = 18). Overall, *n* = 954 adolescent mother–child dyads were included within the subsequent analyses.

### 2.2 Measures

These analyses utilize cross‐sectional data relating to both adolescent mothers and their first‐born children from a range of self‐report questionnaires and standardized developmental assessments.

#### 2.2.1 Adolescent mothers

Sociodemographic characteristics were gathered via self‐report measures. Sociodemographic characteristics include: maternal age, relationship status, housing status, access to resources (i.e., food security), maternal education and/or employment, and maternal violence exposure. Additional sociodemographic characteristics include: maternal age at birth of child (obtained from participant self‐report and corroborated with child dates of birth obtained from child medical records), maternal HIV status (obtained through clinical notes and corroborated by participant or caregiver report on a case‐by‐case basis), perceived social support measured using 8 items from the Medical Outcomes Study (MOS) Social Support Survey (Sherbourne & Stewart, 1991). The MOS Social Support Survey has been previously utilized among adolescents and young people in South Africa (Casale et al., 2019; Filiatreau et al., 2020; West et al., 2019). Experience of any common mental disorder was also measured in the sample (Roberts et al., 2021). Participants were classified as experiencing common mental disorder if they scored above the cut‐off on any of the four mental health symptomatology measures utilized within the study (depressive symptoms [Child Depression Inventory short form ‐ 10 items; CDI‐S; Kovacs & Staff, 2003], anxiety symptoms [Revised Children's Manifest Anxiety Scale ‐ 14 items; RCMAS; Gerard & Reynolds, 1999; Reynolds & Richmond, 1978], Trauma symptoms [Child posttraumatic stress disease‐PTSD checklist ‐ 12 items over four domains of PTSD; Amaya‐Jackson et al., 1995] and suicidality [Mini International Psychiatric Interview for Children and Adolescents −5 items; Sheehan et al., 1997; Sheehan et al., 2010]). The CDI‐S has strong psychometric properties, is well‐validated, and is a widely used measure within South African populations (Suliman, 2002; current sample α = 0.66). The RCMAS has been validated and shows good internal consistency among children and adolescents affected by HIV in South Africa (Boyes & Cluver, 2013; current sample α = 0.86). The Child PTSD checklist has been widely used among adolescents and youth in South Africa (Seedat et al., 2004; Seedat et al., 2000), and the 19‐item scale has been validated within this setting (Boyes et al., 2012; current sample α = 0.79). Globally, the MINI‐KID has been extensively validated, demonstrates good internal consistency and test–retest reliability (Lecrubier et al., 1997; Sheehan et al., 1997; Sheehan et al., 2010; current sample α = 0.91).

#### 2.2.2 Children born to adolescent mothers

Sociodemographic characteristics were collected via adolescent mother/caregiver report. Child sociodemographic characteristics include age (months), biological sex, and childcare attendance. Data were corroborated with data from child medical records where possible. Quartiles (to ensure equal distribution within analyses) were used to explore the relationship between child age (months) and child cognitive development within the sample. Child HIV status was ascertained through clinical notes and corroborated by the adolescent mother/caregiver report on a case‐by‐case basis.

Child cognitive development was assessed across five developmental domains (gross motor skills, fine motor skills, visual reception, expressive language, and receptive language) using the Mullen Scales of Early Learning (Mullen, 1995). Scales were translated into isiXhosa and images were adapted to be contextually relevant, that is, a hairbrush was substituted with a comb. Children were scored across several assessments relating to each domain and raw scores transformed to age standardized t‐scores (range 20–80). T‐scores for four developmental domains—fine motor, visual reception, expressive language, and receptive language—were combined (and converted to age standardized t‐scores utilizing a standardized formula different from the formula to acquire the T‐Scores for the four developmental domains) to create a composite score of generalized cognitive functioning (range 49–155). Only children <=39 months (*n* = 848) were eligible to complete the gross motor skills assessment (based on standard testing procedure; Mullen, 1995). All children completed assessments for all other developmental domains (*n* = 954).

Development scores of children in the sample were compared to those of a normative population (USA derived children developing as expected) outlined in the scoring criteria of the Mullen Scales of Early Learning (Mullen, 1995). Based on this reference population, for the five developmental domains, a mean score of 50 (SD: 10) would be expected, and for the early learning composite score, a mean of 100 (SD:20) would be expected (Mullen, 1995). Descriptive categories of the Mullen Scales of Early Learning were also utilized to identify children at risk for developmental delay. Based on standardized coding (Mullen, 1995), children were classified as scoring *Average and Above* (*t*‐score:40–80) and *Below Average* (*t‐*score:20–39) within the five developmental domains. For the composite score of early learning, based on standardized coding (Mullen, 1995), cut‐offs were prorated for summed T‐scores (visual reception, fine motor, receptive language, expressive language). Children were classified as scoring *Average and above* (summed [standardized] *t‐*score: 85–155) and *Below Average* (summed [standardized] *t‐*score: 49–84). The Mullen Scales of Early Learning have been found to have good psychometric properties, have been adapted for use in South Africa, and have been utilized widely within sub‐Saharan Africa (Boivin et al., 2019; Bornman et al., 2018; Mebrahtu et al., 2020; Milosavljevic et al., 2019).

### 2.3 Statistical analyses

Sample characteristics were using descriptive statistics. Comparisons between the mean cognitive development scores in the sample and the mean score previously identified in the reference population utilized in the development of the Mullen Scales of Early Learning (Mullen, 1995) were undertaken using one‐sample t‐tests. In addition to exploring study data compared to normative data derived from the reference population utilized in the development of the Mullen Scales of Early Learning (Mullen, 1995), additional analyses were undertaken to explore study data in relation to the cognitive development scores (Mullen Scales of Early Learning) of children of adult mothers from the sub‐Saharan African region. To address the absence of reference data relating to the Mullen Scales of Early Learning from the sub‐Saharan African region, a systematic search of the literature was undertaken, and available data was pooled and weighted for comparison. Using a pre‐determined search strategy, studies considered for inclusion within this review were identified through a systematic search of electronic bibliographic databases (see Appendix S1 for further detail on how pooled estimates of Mullen T‐scores from multiple studies were obtained). Where possible, data were extracted according to child HIV status (HIV exposed uninfected/HIV unexposed [classified as developing as expected]). *T*‐tests were used to make comparisons between the mean cognitive development scores in the sample and the mean scores obtained from pooled data from the sub‐Saharan African region. Comparisons stratified according to HIV status (HIV exposed uninfected and HIV unexposed [classified as developing as expected]) were also undertaken to take account of factors that may impact development (e.g., HIV exposure) and, through identifying children classified as developing as expected (HIV unexposed), to establish normative reference data.

To explore age related differences in the sample, analysis of variance (ANOVA) was used to explore T‐scores across each of the developmental domains according to the four age quartiles utilized within the analyses. Where differences were identified, Tukey's HSD post hoc tests were undertaken to further explore differences between groups. Chi‐square tests were utilized to explore the proportion of children in the sample who were identified as being at risk for cognitive delay (scoring *Below Average* on the composite score for early learning) according to the four age quartiles. Age related differences were further examined using linear regression models exploring the cross‐sectional associations between child age (quartiles) and cognitive development scores, and marginal effects were calculated. Covariates were included in multivariable regression models if there were found to be associated with both or either, the predictor and outcome variables (*p* < 0.02; Maldonado & Greenland, 1993; Mickey & Greenland, 1989) or, were identified as relevant within the literature (e.g. social support and likely common mental disorder; Heinze & Dunkler, 2017). The Benjamini‐Hochberg procedure was undertaken to account for multiple testing within regression models (employing a false discovery rate of 10%; Benjamini & Hochberg, 1995). Stata v.15 was used to undertake all analyses.

## 3 RESULTS

### 3.1 Sociodemographic characteristics

Table 1 presents both maternal and first‐born child characteristics. The median age of adolescent mothers at the birth of their first child was 17 years (IQR: 16–18 years). Nearly a quarter, 24.1% (230/954), of adolescent mothers in the sample were living with HIV. Two thirds of mothers reported being in a relationship (622; 65.8%), and 6.7% (64/954) reported having more than one living child. Exactly 71.6% (683/954) reported being food secure, and the majority (92.5%) were accessing social protection in the form of cash grants. The median highest school currently achieved was grade 10 among mothers and 56.9% reported being in either education or employment. Twenty‐seven percent of mothers had been exposed to violence within their communities, and 7.4% had any previous experience of domestic violence. About 85.7% (818/954) of mothers identified as the main caregiver of their children and 25.3% (224/886) of children attended childcare or a creche at least once a week. The average scores of perceived social support among adolescent mothers were high (14; scale 0–14). Within the group of mothers, 12.6% (120/954) were classified as experiencing a common mental disorder (defined as scoring above the cut off points for depression, anxiety, posttraumatic stress, and/or suicidality symptoms). Among the children of adolescent mothers, 48.1% were female, and the average age was 14.5 months (median; interquartile range: 6–28 months).

**TABLE 1:** Sample characteristics of adolescent mothers and their children (*n* = 954).

	Total sample (*n* = 954) *N*(%) M(IQR)
Maternal characteristics	
Current age (years)	18 (17–19)
Age at birth of child (years)	17 (16–18)
11 years	1 (0.1%)
12 years	3 (0.3%)
13 years	15 (1.6%)
14 years	67 (7.0%)
15 years	144 (15.1%)
16 years	207 (21.7%)
17 years	234 (24.5%)
18 years	193 (20.2%)
19 years	90 (9.4%)
Living with HIV	230 (24.1%)
In a relationship	622 (65.8%)
Has more than one child^a^	64 (6.7%)
Food secure	683 (71.6%)
Household cash grant receipt	882 (92.5%)
Number of necessities can afford (0–8)	6 (4–7)
Informal housing	205 (21.9%)
Maternal education – highest grade achieved (0–12)	10 (9–11)
In education or employment	543 (56.9%)
Community violence exposure	258 (27.0%)
Domestic violence exposure	71 (7.4%)
Mother identifies as the primary caregiver of their child	818 (85.7%)
Use of formal childcare (*n* = 886)	224 (25.3%)
Perceived social support (0–14)	14 (14–14)
Any common mental disorder^b^	120 (12.6%)
Child characteristics	
Child biological sex (female)	459 (48.1%)
Child age (months)	14.5 (6–28)

*Note*: Missing data: Use of formal childcare (sample *n* = 886).

Abbreviation: M(IQR), median (interquartile range).

^a^ Report of having two or three live children.

^b^ Common mental disorder (scoring above the cut‐off on one or more screen measure for mental health).

### 3.2 Cognitive development of children born to adolescent mothers compared to the Mullen scales of early learning reference population

**FIGURE 1:**
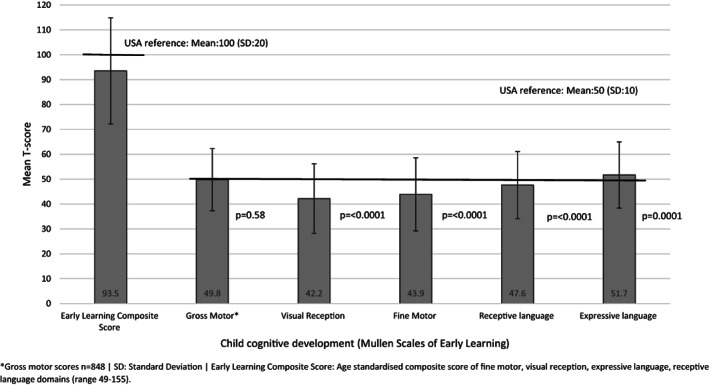
Cognitive development of children born to adolescent mothers compared to the Mullen Scales of Early Learning reference group. *Gross motor scores *n* = 848, SD, Standard Deviation. Early Learning Composite Score: Age standardized composite score of fine motor, visual reception, expressive language, receptive language domains (range 49–155).

Figure 1 presents the cognitive development scores (inclusive of individual domain scores) of children born to adolescent mothers in our study. When compared to the standardized USA reference population utilized within the development of the Mullen Scales of Early Learning, first born children of adolescent mothers performed significantly lower on three of the five domains of cognitive development (visual reception, fine motor, and receptive language), as well as the composite score of early learning (*t =* −9.49, *p* ≤ 0.0001). Similar scores between the sample of children born to adolescent mothers and the USA reference population were identified in relation to gross motor skills scoring, and expressive language scores were found to be higher within the study sample compared to the Mullen reference population (*t =* 3.87, *p* ≤ 0.0001; see Figure 1).

### 3.3 Cognitive development of children of adult mothers in sub‐Saharan Africa compared to the USA derived Mullen scales of early learning reference population

Standardized Mullen T‐scores from nine studies (17 manuscripts) were included within pooled data estimates (see Figure S1). Sample size and standardized Mullen t‐scores were used to weight and calculate pooled data for Mullen t‐scores from the sub‐Saharan African region. Pooled data were also calculated according to whether children were HIV‐exposed, but uninfected or developing as expected (based on sample characteristics detailed within studies included within the pooled data estimates). When compared to the standardized USA reference population utilized within the development of the Mullen Scales of Early Learning, on average children born to adult mothers in the sub‐Saharan African region performed lower on all five domains of cognitive development as well as the composite early learning score (*t* = 13.28, *p* ≤ 0.0001; see Table S2).

### 3.4 Cognitive development of children born to adolescent mothers compared to children of adult mothers in sub‐Saharan Africa

On average, children born to adolescent mothers were older than the children included within the pooled data drawn from sub‐Saharan African studies (18.6 months vs. 17.3 months, *t* = 4.21, *p* ≤ 0.0001). Within the full sample, composite scores of early learning were, on average, similar among children born to adolescent mothers and those children born to adult mothers (obtained from multiple studies [*t* = 0.16, *p* = 0.87; see Table S3 for comparisons on each developmental domain]).

To further explore comparisons between the children of adolescent mothers in the study sample and children of adult mothers (pooled data obtained from multiple studies), data was stratified according to HIV status (HIV‐exposed uninfected, HIV unexposed [classified as developing as expected]). Among children who were HIV‐exposed uninfected/children born to mothers living with HIV (not including children living with HIV), children born to adolescent mothers were on average older than children of adult mothers (24.2 months vs. 22.0 months, *t* = 3.84, *p* = 0.0001). Among this group, composite scores of early learning were similar among children born to adolescent mothers and children of adult mothers (*t* = 1.89, *p* = 0.06; see Table S4 for comparisons on each developmental domain). Among children who were classified as developing as expected, children born to adolescent mothers were on average older than children of adult mothers (16.8 months vs. 13.6 months, *t* = 8.86, *p* ≤ 0.0001). Among this group, children born to adolescent mothers were on average found to have lower composite scores of early learning compared to children born to adult mothers (94.0 vs. 96.6, *t* = −3.39, *p* = 0.0007; see Table S4 for comparisons on each developmental domain, Table 2).

**TABLE 2:** Differences in the cognitive development scores of children born to adolescent mothers according to age bands.

Child cognitive development (Mullen scales of early learning; T‐scores)	Quartiles based on child age (months)				*F*, *p*‐value
	1 (*n* = 256)	2 (*n* = 221)	3 (*n* = 249)	4 (*n* = 228)	
Child age in months M(IQR)	4 (3–5; range:2–6)	10 (8–12; range:7–14)	22 (18–24; range:15–28)	38 (33–46.5; range:29–68)	
Gross motor*	51.2 (9.9)	50.7 (11.5)	51.0 (13.0)	42.5 (15.7)^a,b, c^	16.9, <0.0001
Visual reception	53.1 (8.3)	40.5 (11.0)^a^	33.0 (13.4)^a,b^	41.6 (14.9)^a,c^	118.9, <0.0001
Fine motor	50.3 (8.9)	43.2 (12.7)^a^	41.0 (17.3)^a^	40.5 (15.4)^a^	25.5, <0.0001
Receptive language	58.2 (11.1)	46.8 (11.3)^a^	40.7 (10.2)^a,b^	43.9 (13.9)^a^	108.2, <0.0001
Expressive language	62.8 (8.9)	49.5 (11.1)^a^	43.9 (10.6)^a.b^	49.8 (14.1)^a,c^	127.4, <0.0001
Composite score of early learning^†^	112.2 (13.3)	90.6 (15.8)^a^	80.9 (18.8)^a,b^	88.9 (21.7)^a,c^	145.8, <0.0001
Maternal age at birth of child (years)	16.8	16.7	16.5	16.3	5.1, 0.002

Abbreviation: M(IQR), median (interquartile range).

*Note*: a: Tukey's HSD post hoc test indicates that value is significantly different from quartile 1 (*p* ≤ 0.05). b: Tukey's HSD post hoc test indicates that value is significantly different from quartile 2 (*p* ≤ 0.05). c: Tukey's HSD post hoc test indicates that value is significantly different from quartile 3 (*p* ≤ 0.05).

* Gross motor scores *n* = 848.

^†^ Early Learning Composite Score: Age standardized composite score of fine motor, visual reception, expressive language, receptive language domains (range 49–155).

### 3.5 Differences in the cognitive development of children born to adolescent mothers according to age bands

**FIGURE 2:**
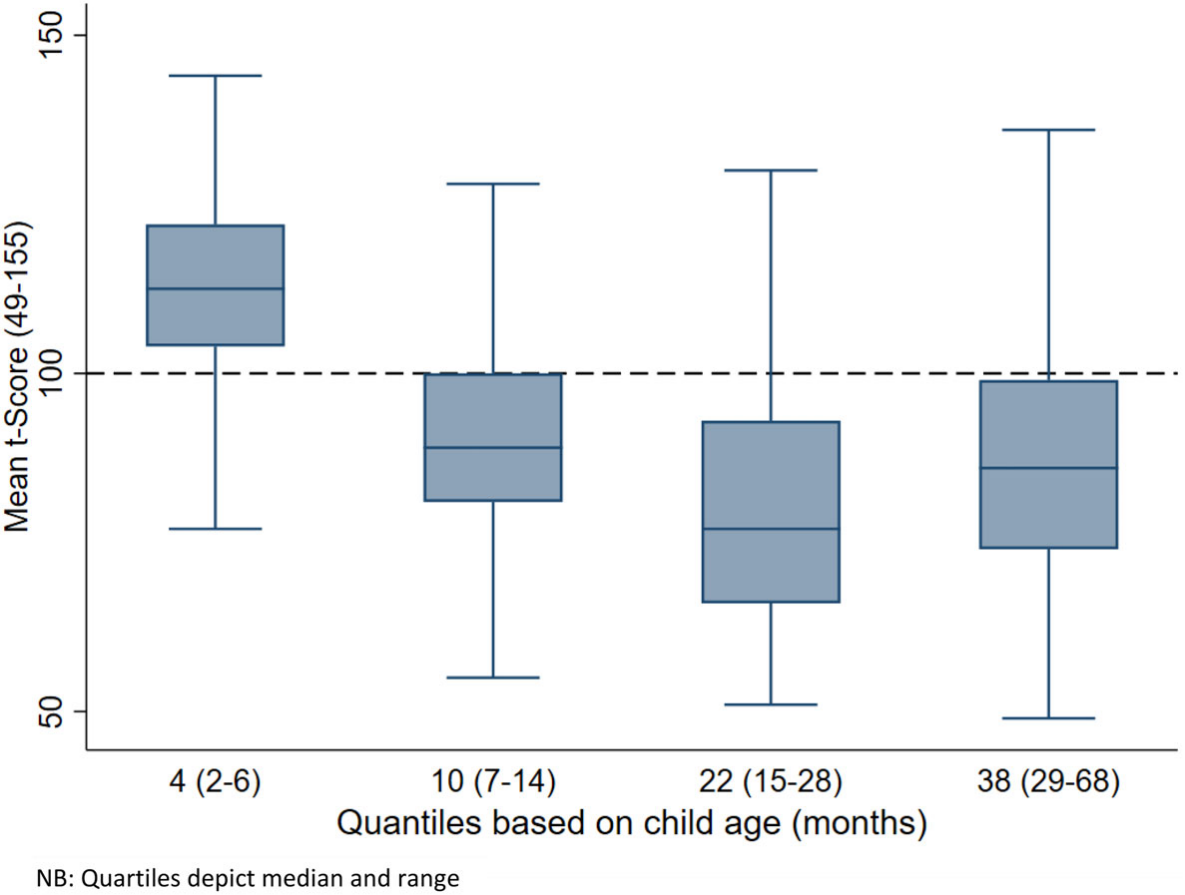
Differences in the cognitive development scores (early years composite score) of children born to adolescent mothers. NB: Quartiles depict median and range.

Table 3 and Figure 2 present child cognitive development scores according to child age, divided into quartiles (months). Differences between quartiles were identified among all the sub‐scales of the Mullen Scales of Early Learning, inclusive of the Early Learning Composite Score, and a decline in the age‐adjusted score was noted in the second quartile, which then remained at a relatively stable level (median child age: 10 months [IQR:8–12, Range:7–14]). Notably, post hoc testing identified that all subsequent quartiles differed from the first quartile (median child age: 4 months [IQR: 3–5, Range: 2–6]).

**TABLE 3:** Linear regression models exploring the association between child age (months; quartiles) and child cognitive development (*n* = 954).

		Composite score of early learning		Gross motor^a^		Visual reception		Fine motor		Receptive language		Expressive language	
Child age quartile	Child age (months; median [IQR])	*B* (95% CI)	*p*	*B* (95% CI)	*p*	*B* (95% CI)	*p*	*B* (95% CI)	*p*	*B* (95% CI)	*p*	*B* (95% CI)	*p*
Model 1.													
1 (*n* = 256)	4 (3–5)	Ref.		Ref.		Ref.		Ref.		Ref.		Ref.	
2 (*n* = 221)	10 (8–12)	‐21.6 (−24.8, −18.4)	**<0.0001**	−0.47 (−2.7, 1.7)	0.67	−12.6 (−14.7, −10.4)	**<0.0001**	−7.1 (−9.6, −4.5)	**<0.0001**	−11.4 (−13.5, −9.3)	**<0.0001**	−13.3 (−15.3, −11.2)	**<0.0001**
3 (*n* = 249)	22 (18–24)	−31.3 (−34.4, −28.2)	**<0.0001**	−0.26 (−2.4, 1.9)	0.81	−20.1 (−22.2, −18.0)	**<0.0001**	−9.3 (−11.7, −6.8)	**<0.0001**	−17.5 (−19.6, −15.5)	**<0.0001**	−18.9 (−20.9, −16.9)	**<0.0001**
4 (*n* = 228)	38 (33–46.5)	−23.3 (−26.4, −20.1)	**<0.0001**	−8.7 (−11.4, −6.1)	**<0.0001**	−11.4 (−13.6, −9.3)	**<0.0001**	−9.8 (−12.3, −7.3)	**<0.0001**	−14.3 (−16.4, −12.2)	**<0.0001**	−13.0 (−15.0, −10.9	**<0.0001**
Adjusted *R* ^2^		0.315		0.053		0.271		0.072		0.252		0.285	
Model 2.													
1 (*n* = 256)	4 (3–5)	Ref.		Ref.		Ref.		Ref.		Ref.		Ref.	
2 (*n* = 221)	10 (8–12)	−22.3 (−25.6, −19.0)	**<0.0001**	−1.2 (−3.5, 1.1)	0.31	−12.9 (−15.1, 10.7)	**<0.0001**	−7.8 (−10.4, −5.1)	**<0.0001**	−11.3 (−13.5, −9.1)	**<0.0001**	−13.8 (−15.9, −11.7)	**<0.0001**
3 (*n* = 249)	22 (18–24)	−33.6 (−37.0, −30.3)	**<0.0001**	−1.2 (−3.5, 1.2)	0.33	−21.4 (−23.7, −19.2)	**<0.0001**	−10.9 (−13.6, −8.2)	**<0.0001**	−18.5 (−20.8, −16.3)	**<0.0001**	−19.9 (−22.0, −17.7)	**<0.0001**
4 (*n* = 228)	38 (33–46.5)	−27.0 (−30.9, −23.0)	**<0.0001**	−9.4 (−12.6, −6.3)	**<0.0001**	−13.4 (−16.1, −10.7)	**<0.0001**	−11.5 (−14.7, −8.3)	**<0.0001**	−16.2 (−18.9, −13.6)	**<0.0001**	−15.1 (−17.7, −12.5)	**<0.0001**
Adjusted *R* ^2^		0.335		0.653		0.298		0.084		0.262		0.299	

*Note*: Model 1. Univariate analyses. Model 2. Multivariable analyses. Covariates included with Model 2: maternal HIV status (living with HIV), maternal mental health (any common mental disorder), maternal age at birth of child (years), maternal relationship status (in a relationship), food security (food secure), maternal education (highest school grade achieved), number of basic necessities household can afford, child sibling status (has siblings), maternal cash grant receipt (yes), maternal community violence exposure (no), maternal domestic violence exposure (yes), maternal perceived social support, maternal work or education status (in work or education), child early child development programme attendance (yes), child biological sex (female).

Bold values indicate a statistical significance of *p* = <0.05.

^a^ Gross motor scores *n* = 848.

To explore age related differences further, the proportions of children scoring *Below Average* and *Average and above* (based on the Mullen reference group) were examined. For composite scores of early learning, group differences were identified across age quartiles (X^2^ = 203.0, *p* ≤ 0.0001). About 3.1% of children were classified as scoring *Below* Average in the first quartile (Median child age:4 months [IQR:3–5; Range:2–6]), 36.7% scored *Below Average* in the second quartile (Median child age:10 months [IQR: 8–12, Range:7–14]), 62.3% scored *Below Average* in the third quartile (Median child age:22 months [IQR:18–24, Range:15–28]) and, 46.1% scored *Below Average* in the fourth quartile (Median child age:38 months [IQR:33–46.5], Range:29–68).

**FIGURE 3:**
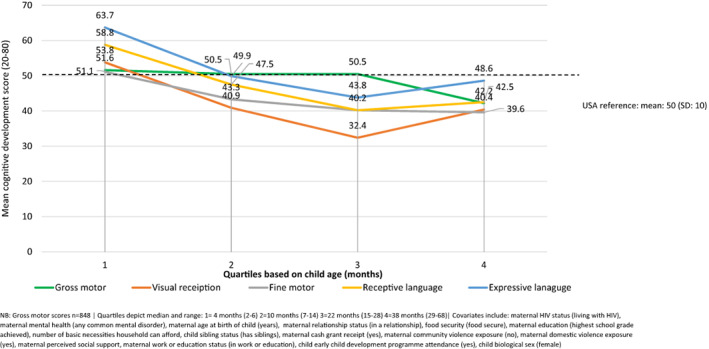
Adjusted mean cognitive development scores obtained for children born to adolescent mothers stratified according to child age (months; quartiles, *n* = 954). NB: Gross motor scores *n* = 848. Quartiles depict median and range: 1 = 4 months (2–6) 2 = 10 months (7–14) 3 = 22 months (15–28) 4 = 38 months (29–68). Covariates include: maternal HIV status (living with HIV), maternal mental health (any common mental disorder), maternal age at birth of child (years), maternal relationship status (in a relationship), food security (food secure), maternal education (highest school grade achieved), number of basic necessities household can afford, child sibling status (has siblings), maternal cash grant receipt (yes), maternal community violence exposure (no), maternal domestic violence exposure (yes), maternal perceived social support, maternal work or education status (in work or education), child early child development programme attendance (yes), child biological sex (female).

Table 3 presents a series of linear regression models exploring the cross‐sectional association between child age (quartiles) and child cognitive development scores. After adjusting for covariates, similar patterns of development scores were identified across all domains of the Mullen Scales of Early Learning, apart from the gross motor domain, and in the composite scores of early learning. Compared to the first age quartile (median child age: 4 months [IQR: 3–5, Range:2–6]), children in the second age quartile (median child age: 10 months [IQR: 8–12, Range:7–14]) were scoring lower on cognitive assessments (*B* = −21.6 [95% CI: −24.8, −18.4], *p* ≤ 0.0001). Scores on cognitive domains reduced further by the third age quartile (median child age: 22 months [IQR: 18–24, Range: 15–28]; *B* = −31.3 [95% CI: −34.4, −28.2], *p* ≤ 0.0001) and improved slightly (compared to the third age quartile) by the fourth age quartile (median child age: 38 months [IQR: 33–46.5, Range: 29–68]; *B* = −23.2 [95% CI: −26.4, −20.1], *p* = 0.0001; Adjusted *R* ^2^ = 0.315). For gross motor skill scores, there was no difference identified among children in the second age quartile (median child age: 10 months [IQR: 8–12, Range:7–14]) compared to the first age quartile (median child age: 4 months [IQR:3–5, Range:2–6]), nor the third age quartile (median child age: 22 months [IQR: 18–24, Range: 15–28]). However, compared to children in the first age quartile (median child age: 4 months [IQR: 3–5, Range:2–6]), children in the fourth age quartile (median child age: 38 months [IQR: 33–46.5, Range: 29–68]) scored lower on the gross motor skills domain (*B* = −8.7 [95% CI: −11.4, −6.1], *p* ≤ 0.0001; Adjusted *R* ^2^ = 0.05). These associations remained significant when using the Benjamini‐Hochberg procedure for multiple testing with a false discovery rate of 10%. Figure 3 presents the adjusted mean scores (set to the average levels of all included covariates) for each of the developmental domains according to child age quartiles, reflecting the regression models above.

### 3.6 Differences in the cognitive development of children born to adolescent mothers according to maternal age

The age of mothers at the birth of their first child ranged from 11 to 19 years (see Table 1). Table 4 presents a series of linear regression models exploring the cross‐sectional association between maternal age at the birth of their child and child cognitive development scores. While univariate analyses identified a trend between maternal age at the birth of their child and child cognitive development, with older maternal age possibly associated with higher composite scores of early learning (B = 0.91 [95%CI: 0.009, 1.82], *p* = 0.05; Adjusted *R* ^2^ = 0.003), this relationship was not maintained in the multivariate analyses (B = 0.61 [95% CI: −0.51, 1.74], *p* = 0.29; Adjusted *R* ^2^ = 0.02). Maternal age at the birth of their child was similarly found to be likely associated with child fine motor skills scores after adjusting for covariates (B = 0.84 [95% CI: 0.06, 1.62], *p* = 0.04; Adjusted *R* ^2^ = 0.005). However, this association did not remain significant when using the Benjamini‐Hochberg procedure for multiple testing with a false discovery rate of 10%. Maternal age at the birth of their first child was not found to be associated with any other developmental domains.

**TABLE 4:** Linear regression models exploring the association between maternal age at the birth of their child (years) and child cognitive development (*n* = 954).

	Composite score of early learning		Gross motor^a^		Visual reception		Fine motor		Receptive language		Expressive language	
	*B* (95% CI)	*p*	*B* (95% CI)	*p*	*B* (95% CI)	*p*	*B* (95% CI)	*p*	*B* (95% CI)	*p*	*B* (95% CI)	*p*
Model 1.												
Maternal age at birth (years)	0.91 (0.009, 1.82)	0.05	0.08 (−0.50, 0.66)	0.79	0.34 (−0.26, 0.95)	0.26	0.65 (0.03, 1.28)	**0.04**	0.44 (−0.13, 1.02)	0.13	0.53 (−0.04, 1.10)	0.07
Adjusted *R* ^2^	0.003		0.001		0.001		0.003		0.001		0.003	
Model 2.												
Maternal age at birth (years)	0.61 (−0.51, 1.74)	0.29	0.20 (−0.53, 0.93)	0.59	−0.04 (−0.79, 0.71)	0.92	0.84 (0.06, 1.62)	**0.04**	0.37 (−0.34, 1.08)	0.30	0.17 (−0.54, 0.88)	0.64
Adjusted *R* ^2^	0.018		0.021		0.015		0.005		0.019		0.016	

*Note*: Model 1. Univariate analyses. Model 2. Multivariable analyses. Covariates included with Model 2: maternal HIV status (living with HIV), maternal mental health (any common mental disorder), maternal relationship status (in a relationship), food security (food secure), maternal education (highest school grade achieved), number of basic necessities household can afford, child sibling status (has siblings), maternal cash grant receipt (yes), maternal community violence exposure (no), maternal domestic violence exposure (yes), maternal perceived social support, maternal work or education status (in work or education), child early child development programme attendance (yes), child biological sex (female).

Bold values indicate a statistical significance of *p* = <0.05.

^a^ Gross motor scores *n* = 848.

## 4 DISCUSSION

This is the first known exploration of cognitive development among children born to adolescent mothers in South Africa, compared to normative reference values on a standardized validated cognitive development performance inventory, and the first known comparison of development according to age bands among this group. Overall, children in the sample (0–68 months) obtained lower development scores compared to the reference population utilized within the Mullen Scales of Early Learning, however, within individual domains, no difference was identified on the gross motor domain, and children in the sample were overall found to score slightly higher on the expressive language domain compared to the reference population. Some writers argue that cultural factors may limit the utility of USA‐based validated scales in other settings, while others have shown the universality of such measures and used these scales efficiently in varied global contexts. This study therefore also found that, compared to children of adult mothers in the sub‐Saharan African region, children born to adolescent mothers (classified as developing as expected) were found to have lower overall cognitive development scores. Yet, within individual domains, gross motor scores were similar among children of adolescent mothers and adult mothers, and children born to adolescent mothers on average had slightly higher receptive language scores compared to children born to adult mothers in the region. Differences according to age were identified within the sample. Cognitive development scores were found to be similar among younger children within the sample compared to the reference population; however, older children in the sample were found to have lower development scores when compared to the reference population. Analyses also identified a potentially complex non‐linear relationship between maternal age and child cognitive development scores. Findings from this study support previous research identifying children within low‐ and middle‐income countries as being at risk for cognitive adversity (Engle et al., 2011; Grantham‐McGregor et al., 2007; Hunt & Tomlinson, 2018; Milosavljevic et al., 2019; Walker et al., 2007) and address an evidence gap regarding the cognitive development of children of adolescent mothers within the sub‐Saharan African region (including those affected by HIV).

### 4.1 Cognitive performance of children born to adolescent mothers

Differences in the cognitive performance scores of the sample and the normative Mullen reference data (Mullen, 1995) were found within overall cognitive development and the majority of the individual subscales (apart from gross motor skills). Similarity among gross motor skill scores is possibly due to the gross motor skills sub‐scale assessing broader measures of development (e.g., sitting, standing, walking, and running), for which there may be less variation in the sample, particularly given that the gross motor scales was only undertaken among children <39 months. On average, expressive language scores in the sample were slightly elevated compared to those in the Mullen Scales of Early Learning reference data. There is a strong body of evidence that has identified an association between motor skill development and expressive language skills. As children develop new motor skills, the way they interact with their environment and as such their ability to communicate changes, often increasing (Walle & Campos, 2014). This hypothesis is supported by the overall average scores within the sample being similar in the gross motor and expressive language sub‐scales. Differences in sub‐scale scores highlight potentially different developmental trajectories of children born to adolescent mothers in South Africa and the normative reference population of children of adult mothers in the USA, possibly due to contextual factors, such as service access. The high prevalence of HIV within the region and, thereafter, the high prevalence of HIV exposed‐uninfected children may be a consideration and a contributor to varying cognitive development trajectories.

Given the lack of reference data from the sub‐Saharan African region and thus the lack of culturally and geographically relevant reference data, the available literature utilizing Mullen T‐scores was summarized to be able to make comparisons between the development of children born to adolescent mothers and those born to adult mothers in the region. Differences in the cognitive performance scores of children born to adolescent mothers (classified as developing as expected) and pooled data drawn from children born to adult mothers in the sub‐Saharan African region were also identified. Like the findings relating to the USA derived reference data, gross motor scores were similar among the groups. Similarly, receptive language scores in the sample were slightly elevated compared to the data from children born to adult mothers in the region, again highlighting potentially different developmental trajectories for children born to adolescent mothers compared to children of adult mothers. Overall lower cognitive development scores among children born to adolescent mothers compared to children of adult mothers in the region also highlight a potential risk of developmental delay among children born to adolescent mothers. It should also be noted that children born to adolescent mothers were slightly older than children of adult mothers. Lower developmental scores were linked to increasing age in the broader findings of this study, thus findings relating to children of adolescent mothers being older than the children of adult mothers (obtained from pooled data) further support the notion of a potential risk of developmental delay among children born to adolescent mothers in South Africa. As such, there is a potential need for support to promote the development of children born to adolescent mothers.

### 4.2 Differences in cognitive performance according to age

Within an age stratified sample, differences in development scores began to be observed by the second quartile (Median 10 months; IQR: 8–12) and continued to be evident through the older aged children within the sample, within both composite scoring and scoring on individual subscales. This finding has potentially monumental practical, clinical, and public health implications as it highlights that early intervention may be required to support the development of children within this sample. Optimal child development should continue along a trajectory; however, the complex interplay between biological and environmental factors may expose vulnerabilities for some children (Maggi et al., 2010). Such findings support previous literature identifying reduced cognition among children living within low resourced settings within their first 12 months of age (Hamadani et al., 2014; Koura et al., 2013; Milosavljevic et al., 2019), as they begin to be increasingly exposed to environmental factors. Like a recent study exploring the cognitive development of children in The Gambia, with rising age (up until 24 months; quartiles 2 and 3), the proportion of children classified as scoring *Below Average* (on the composite scores of early learning) and, therefore, were at risk for cognitive delay, continued to rise. This proportion is reduced for older children (median child age: 38 months [IQR: 33–46.5]), perhaps indicative exposure to increased environmental stimuli or differing skill development trajectories.

However, variation in individual subscales should be noted, that is, visual reception scores were much lower than other domains by the second age quartile, and while language and visual reception t‐scores were higher among those children in the fourth age quartile (median child age: 38 months [IQR: 22–46.5]), motor skill scores continued to decline, perhaps suggesting that on average motor skills are developing at a slower rate than expected. Such differences in the onset of decline among differing developmental domains may be due to the differing timings in which such developmental milestones are expected to ensue (Milosavljevic et al., 2019). Furthermore, findings support literature identifying disparities within perceptual domains as an early indication of adverse child development outcomes. As such, findings highlight potential risk for cognitive delay among this sample (Milosavljevic et al., 2019) and a core developmental period where risk for cognitive delay may emerge within such settings.

This lower scoring is likely due to the potential developmental challenges faced by children living in a context of high poverty, high HIV burden, and challenges of adolescent parenthood (e.g., poor food security or nutrition, linked to poverty, may have implications for growth and stunting) (Akombi et al., 2017). In addition, while those children known to be living with HIV were excluded from analyses, almost a quarter of children within the sample were potentially HIV exposed. There is a body of literature detailing the association between HIV exposure and child developmental challenges (Chaudhury et al., 2017; Kerr et al., 2014). Furthermore, differences may be explained by differing growth trajectories of children within South Africa and those within the USA reference group (e.g., children learning different cognitive skills at different rates or in different orders). Differences between the children in the sample and the reference group may also be due to some of the items administered within the Mullen scales potentially not being relevant to the South African setting. However, it should be noted that the Mullen Scales of Early Learning have been utilized extensively within the study of child development within South Africa and the sub‐Saharan African region (Bornman et al., 2018).

All mothers within the sample were adolescents during the birth of their child; however, the experiences of a younger adolescent mother may differ compared to an older adolescent mother. Analyses allude to a complex non‐linear relationship between maternal age at the birth of their child and child cognitive development scores while an association was identified in univariate analyses, this association was not retained in multivariate analyses. Such findings support the notion that multiple factors beyond child and maternal age may impact child cognitive development and that broader contextual factors may play a role within the development of children in this setting (e.g., wider family support). Further research is required to explore mediating and moderating factors in the relationship between maternal age and child cognitive development. It would be important, as a next step, to consider additional factors associated with adolescent parenthood that may contribute to child stimulation and growth, such as school return, alternative care arrangements, the involvement of multiple adults (including grandparents) in child‐care and access pathways to quality childcare services and early child development (ECD) resources.

This study highlights the potential need for intervention to bolster child development outcomes in this population. High quality care and programmatic responses, such as book sharing have previously been found to improve child development outcomes, providing a promising avenue for intervention (Vally et al., 2015). Parenting interventions have also been found to be particularly successful in bolstering cognitive (inclusive of language and motor skills) development within such settings, and, magnified effects have been identified among interventions that include a component on responsive caregiving (Jeong et al., 2021). While there has been a recent shift towards programming for adolescent mothers affected by HIV and their children (Toska et al., 2020), the effectiveness of interventions for adolescents living with HIV and their children remains largely unknown. Interventions may require tailoring to meet the specific needs of adolescent mothers and their children in settings of high HIV prevalence.

### 4.3 Limitations

Study limitations should be considered in the interpretation of findings from these analyses. First, data within these analyses is cross‐sectional, and thus, the comparisons between age groups do not represent a longitudinal analysis of development trajectories, but instead a comparison of different children of differing ages. Second, the Mullen Scales of Early Learning were developed in and use a reference group from the USA (Mullen, 1995). Given the absence of a comparison group for the current sample, analyses within this study utilized data from the USA reference population. The validity of the USA reference population for examining broader samples has previously been criticized (e.g., differences in context) (Akshoomoff, 2006; Faruk et al., 2020). Yet, to develop an understanding of how populations of interest are faring in the absence of reference data for broader populations, such as this sample, such comparisons remain the current analytical practice (Milosavljevic et al., 2019). It should be noted that the Mullen Scales of Early Learning have been utilized extensively throughout sub‐Saharan Africa (Bornman et al., 2018), inclusive of South Africa (Bornman et al., 2018), and that an independent assessment of child cognitive development is preferable to caregiver reporting. The Mullen Scales of Early Learning were administered by trained local data collectors and extensively piloted with children from the population of interest to ensure that items were understood and appropriate for children within the sample. To address the lack of a locally derived normative reference population, data from the sub‐Saharan African region were systematically summarized and comparisons with the sample were made. It should be noted that the summary data did not include data from South Africa due to the variability in scoring of the Mullen Scales utilized within previous studies. Third, due to the age distribution of children within the sample, age quartiles were utilized within the exploration of cognitive development. Future studies with equally distributed samples may benefit from repeating analyses based on age categories rather than quartiles to pinpoint key developmental periods. Fourthly, the timing relating to the use of the Mullen Scales of Early Learning should remain a consideration (e.g., differing contextual factors in previous studies utilizing the Mullen Scales of Early Learning compared to current data may have implications for child development scoring). Finally, it was beyond the scope of this study to explore the profile of children living with HIV.

Future studies are encouraged to utilize longitudinal data to explore changes in child cognitive development throughout the development course of children born to adolescent mothers. Well‐validated scales, even from different populations, allow for detailed understanding, and until there is equivalent local resources, international measures do seem to have utility, and regional normative data can be collected for comparison purposes. Additional avenues of research include further exploring the cognitive development according to maternal HIV status, the cognitive profiles of second‐ and third‐born children of adolescent mothers, how adolescent mothers and their children compare to children of adult populations within South Africa, as well as pathways/factors promoting the cognitive development of children born to adolescent mothers within such contexts.

## 5 CONCLUSIONS

These findings address a critical evidence gap regarding the cognitive development of children born to adolescent mothers and identify a potential need for intervention to promote child cognitive development within early childhood for this group. Children born to adolescent mothers in this setting were found to score lower than the reference population, and, while the younger aged children in the sample were comparable to the reference population, older children were found to score lower across development domains, identifying a core developmental period that may be amenable to intervention. Findings also identify a potential risk of developmental delay among children born to adolescent mothers compared to children of adult mothers in the sub‐Saharan African region. Existing interventions for the promotion of child cognitive development may be appropriate, however, adaptation may be required for the specific needs of children born to adolescent mothers affected by HIV within the South African context.

## AUTHOR CONTRIBUTIONS

**Colette Smith:** Conceptualization; formal analysis; methodology; supervision; visualization; writing – original draft; writing – review and editing. **Elona Toska:** Conceptualization; data curation; funding acquisition; investigation; methodology; project administration; supervision; visualization; writing – review and editing. **Lucie Cluver:** Conceptualization; data curation; funding acquisition; investigation; methodology; project administration; supervision; visualization; writing – review and editing. **Camille Wittesaele:** Data curation; investigation; methodology; project administration; writing – review and editing. **Nontokozo Langwenya:** Data curation; project administration; writing – review and editing. **Yulia Shenderovich:** Formal analysis; investigation; methodology; writing – review and editing. **Wylene Saal:** Data curation; investigation; methodology; writing – review and editing. **Janina Jochim:** Data curation; formal analysis; methodology; project administration; writing – review and editing. **Jenny Chen‐Charles:** Investigation; project administration; writing – review and editing. **Marguerite Marlow:** Methodology; project administration; resources; writing – review and editing. **Lorraine Sherr:** Conceptualization; formal analysis; funding acquisition; investigation; methodology; project administration; supervision; visualization; writing – original draft; writing – review and editing.

## FUNDING INFORMATION

The HEY BABY study was jointly funded by the UK Medical Research Council (MRC) and the UK Department for International Development (DFID) under the MRC/DFID Concordat agreement, and by the Department of Health Social Care (DHSC) through its National Institutes of Health Research (NIHR) [MR/R022372/1]; the European Research Council (ERC) under the European Union's Horizon 2020 research and innovation programme (no. 771468); the UKRI GCRF Accelerating Achievement for Africa's Adolescents (Accelerate) Hub (Grant Ref: ES/S008101/1); the Fogarty International Center, National Institute on Mental Health, National Institutes of Health under Award Number K43TW011434, the content is solely the responsibility of the authors and does not represent the official views of the National Institutes of Health; a CIPHER grant from International AIDS Society [2018/625‐TOS], the views expressed do not necessarily reflect the official policies of the International AIDS society; Research England [0005218], the Leverhulme Trust (PLP‐2014‐0950), HelpAge in conjunction with NORAD Sweden, the Oak Foundation (OFIL‐20‐057) and UNICEF Eastern and Southern Africa Regional Office (UNICEF‐ESARO). KJSR is supported by an Economic Social Research Council PhD studentship through UBEL‐DTP (UK). YS is supported by DECIPHer and the Wolfson Centre for Young People's Mental Health. DECIPHer is funded by the Welsh Government through Health and Care Research Wales. The Wolfson Centre for Young People's Mental Health has been established with support from the Wolfson Foundation.

## CONFLICT OF INTEREST STATEMENT

The authors declare no conflict of interest.

### PEER REVIEW

The peer review history for this article is available at https://publons.com/publon/10.1002/icd.2408.

## Supporting information


**Appendix S1.** Supporting Information

## Data Availability

Prospective users, policymakers/government agencies/researchers (internal/external) will be required to contact the study team to discuss and plan the use of data. Research data will be available on request subject to participant consent and having completed all necessary documentation. All data requests should be sent to the Principal Investigators.
