# Supplementary material for: Exploring the cognitive development of children born to adolescent mothers in South Africa
Source: Infant Child Dev. 2023 Mar 7;32(3):e2408. doi: 10.1002/icd.2408 (PMC10909423; doi:10.1002/icd.2408)
Supplement: Supplementary file 1 — Appendix S1. Supporting Information [file ICD-32-e2408-s001.docx]

**Supplementary material**

**Pooled Mullen Scales of Early Learning t-scores from sub-Saharan Africa**

To provide an overview of Mullen t-scores from the sub-Saharan African region a systematic review of the literature was undertaken.

**Inclusion and exclusion criteria**

Studies were included if Mullen t-scores (standardised scores) were clearly identifiable, and data was drawn from a sub-Saharan African population. Studies were excluded if child age and t-scores (for all domains of the Mullen Scales of Early Learning) we not identifiable, children in the sample were solely living with HIV or, were identified as having a developmental disability. When manuscripts were identified as being drawn from the same study, the manuscript with the largest/most representative sample was included within the pooled data. When samples included data stratified by HIV status (living with HIV, HIV exposed uninfected and, HIV unexposed) data was extracted for children who were HIV exposed uninfected and/or HIV unexposed.

**Search strategy and selection criteria**

Using a pre-determined search strategy, studies considered for inclusion within this review were identified through a systematic search of electronic bibliographic databases. Databases searched included: PsycINFO (1806-present) and, PubMed (including Medline, 1966-present). Search terms included all sub-Saharan African countries and “Mullen”. Citations within manuscripts selected for full-text review were also used to identify additional studies for inclusion with the pooled data. Database searches were undertaken in August 2021.

**Data extraction**

All titles and abstracts identified were examined for relevance. Full manuscripts of any potentially relevant studies were obtained and assessed for inclusion based on the above inclusion criteria. Information extracted from relevant manuscript included publication detail, geographical location, sample details, methodology, details of measurement and standardised t-scores.

**Data synthesis**

Sample size and standardised Mullen t-scores were used to weight and calculate pooled data for Mullen t-scores from the sub-Saharan African region. Pooled data were also calculated according to whether children were HIV-exposed uninfected or developing as expected (based on sample characteristics detailed within studies included within the pooled data estimates).

**Pooled Mullen Scales of Early Learning t-scores – Results**

Standardised Mullen t-scores from 9 studies (17 manuscripts) were included within pooled data estimates (see Supplementary Figure 1). Supplementary Table 2 details the Mullen t-scores obtained from all included studies as well as pooled data (based on means [SD]). All studies included of children born to adult mothers.

**Supplementary Table 1. Cognitive development scores of children born to adolescent mothers compared to the Mullen Scales of Early Learning reference group**

| **Child cognitive development (Mullen Scales of Early Learning; T-Scores)** | ***Mean (SD)*** | | ***t, p*-value** |
| --- | --- | --- | --- |
|  | **Children born to adolescent mothers (n=954)** | **Mullen Scales reference group (USA population)** |  |
| Composite score of early learning^a^ | 93.5 (21.3) | 100 (20) | **-9.49, <0.0001** |
| Gross motor* | 49.8 (12.5) | 50 (10) | -0.56, 0.58 |
| Visual reception | 42.2 (14.0) | 50 (10) | **-17.06, <0.0001** |
| Fine motor | 43.9 (14.7) | 50 (10) | **-12.86, <0.0001** |
| Receptive language | 47.6 (13.5) | 50 (10) | **-5.62, <0.0001** |
| Expressive language | 51.7 (13.3) | 50 (10) | **3.87, 0.0001** |

^a^Early Learning Composite Score: Age standardised composite score of fine motor, visual reception, expressive language, receptive language domains (range 49-155) |*Gross motor scores n=848

**Supplementary Table 2: Studies identified for inclusion within pooled data estimates from the sub-Saharan African region and calculated pooled Mullen Scales of Early Learning t-scores**

|  | **Author (year)** | **Country** | **Total sample (n)** | **Child age (months)** | **Study population (children)** | **Composite score of early learning t-score^a^** | **Gross motor t-score** | **Visual reception t-score** | **Fine motor t-score** | **Receptive language t-score** | **Expressive language t-score** |
| --- | --- | --- | --- | --- | --- | --- | --- | --- | --- | --- | --- |
| 1 | Mebrahtu et al. 2020^1^ /Mebrahtu et al 2018^2^ /Mebrahtu 2020^3^ /Mebrahtu et al. 2019^4^ | Zimbabwe | 562 | 11.9 (6.5) | HEU | 102.5 (17.9) | 50.5 (10.9) | 53.1 (12.7) | 50.8 (11.4) | 47.7 (11.5) | 52.9 (10.7) |
| 2 | Kashala-Abotnes et al. 2018^5^ | Democratic Republic of Congo | 114 | 31.0 (4.0) | Developing as expected | 79.4 (16.0) | 41.1 (11.3) | 37.4 (11.3) | 37.2 (13.2) | 41.7 (10.3) | 38.4 (9.8) |
| 3 | Familiar et al. 2016^6^~ | Uganda | 164 | 34.1 (4.4) | HEU | 71.7 (10.9) | 26.3 (3.1) | 29.9 (9.1) | 35.0 (8.5) | 38.3 (8.2) | 35.7 (9.7) |
| 4 | Chhaya et al. 2018^7^ | Uganda | 31 | 9.1 (1.9) | HEU | 96.4 (13.2) | 51.4 (9.2) | 48.4 (10.9) | 48.5 (11.7) | 44.0 (9.7) | 51.7 (8.6) |
| 5 | Boivin et al. 2021^8^ /Garrison et al. 2021^9^ /Mireku et al. 2020^10^  /Mireku et al. 2015^11^ / Bodeau-Livinec et al. 2019^12^ /Koura et al 2013^13^ | Benin | 747 | 12.1 (0.7) | Developing as expected (mothers recruited to a trial comparing preventative treatments for malaria and helminth infection) | 98.7 (11.2) | 51.3 (14.1) | 50.5 (10.4) | 50.2 (9.5) | 46.6 (6.7) | 49.3 (10.4) |
| 6 | Boivin et al. 2017^14^ | Uganda | 221 | 34.4 (5.1) | HEU | 70.8 (11.2) | 26.5 (3.3) | 29.6 (9.0) | 33.9 (8.6) | 38.1 (8.3) | 35.6 (9.9) |
| 7 | Boivin et al. 2013^15^ | Uganda | 119 | 33.6 (4.0) | HEU | 85.1 (16.2) | 37.1 (9.8) | 39.6 (11.6) | 41.1 (11.2) | 46.2 (9.6) | 41.2 (10.4) |
| 8 | Boivin et al. 2019^16^^* | Uganda & Malawi | 374 | 12.0 | Developing as expected (HUU) | 95.2 (14.5) | 47.9 (10.7) | 49.2 (10.0) | 50.6 (8.9) | 45.0 (9.3) | 44.7 (10.1) |
| 9 | Familiar et al 2018^17^^ | Uganda | 149 | 12.0 | Developing as expected (HUU) | 102.8 (15.3) | 52.6 (11.7) | 52.9 (8.9) | 54.4 (12.9) | 46.7 (7.0) | 50.8 (10.9) |
| **Pooled Data** | | | | | | | | | | | |
|  | **-** | **-** | **2481** | **17.3 (2.8)** | **Total sample** | **93.4 (14.6)** | **45.7 (10.6)** | **46.7 (10.7)** | **47.0 (9.7)** | **44.8 (7.7)** | **46.4 (10.3)** |
|  |  | **-** | **1384** | **13.6 (0.7)** | **Developing as expected** | **96.6 (14.2)** | **49.7 (12.7)** | **49.3 (10.2)** | **49.7 (10.0)** | **45.8 (7.7)** | **47.3 (10.3)** |
|  | **-** | **-** | **1097** | **22.0 (5.5)** | **HEU** | **89.4 (15.2)** | **40.6 (8.0)** | **43.3 (11.2)** | **38.9 (10.3)** | **39.2 (8.5)** | **39.5 (10.1)** |

All values are Mean (SD) unless otherwise stated |When manuscripts detailed data from the same study the largest/most relevant sample were selected for inclusion (first study listed) | HEU: HIV-exposed uninfected | HUU: HIV-unexposed |~data for children living with HIV excluded from pooled data | ^only HUU data reported |*only data for children at 12 months of age included | ^a^Early Learning Composite Score: Age standardised composite score of fine motor, visual reception, expressive language, receptive language domains (range 49-155)

^1^ Mebrahtu H, Sherr L, Simms V, Weiss HA, Rehman AM, Ndlovu P, Cowan FM. Effects of maternal suicidal ideation on child cognitive development: A longitudinal analysis. AIDS and Behavior. 2020 Aug;24(8):2421-9.

^2^ Mebrahtu H, Simms V, Chingono R, Mupambireyi Z, Weiss HA, Ndlovu P, Malaba R, Cowan FM, Sherr L. Postpartum maternal mental health is associated with cognitive development of HIV-exposed infants in Zimbabwe: a cross-sectional study. AIDS care. 2018 Jun 20;30(sup2):74-82.

^3^ Mebrahtu H, Sherr L, Simms V, Weiss HA, Chingono R, Rehman AM, Ndlovu P, Cowan FM. The impact of common mental disorders among caregivers living with HIV on child cognitive development in Zimbabwe. AIDS care. 2020 May 13;32(sup2):198-205.

^4^ Mebrahtu H, Simms V, Mupambireyi Z, Rehman AM, Chingono R, Matsikire E, Malaba R, Weiss HA, Ndlovu P, Cowan FM, Sherr L. Effects of parenting classes and economic strengthening for caregivers on the cognition of HIV-exposed infants: a pragmatic cluster randomised controlled trial in rural Zimbabwe. BMJ global health. 2019 Sep 1;4(5):e001651.

^5^ Kashala-Abotnes, E., Sombo, M. T., Okitundu, D. L., Kunyu, M., Bumoko Makila-Mabe, G., Tylleskär, T., ... & Boivin, M. J. (2018). Dietary cyanogen exposure and early child neurodevelopment: An observational study from the Democratic Republic of Congo. *PloS one*, *13*(4), e0193261.

^6^ Familiar I, Nakasujja N, Bass J, Sikorskii A, Murray SM, Ruisenor-Escudero H, Bangirana P, Opoka R, Boivin MJ. Caregivers' depressive symptoms and parent-report of child executive function among young children in Uganda. Learning and individual differences. 2016 Feb 1;46:17-24.

^7^ Chhaya R, Weiss J, Seffren V, Sikorskii A, Winke PM, Ojuka JC, Boivin MJ. The feasibility of an automated eye-tracking-modified Fagan test of memory for human faces in younger Ugandan HIV-exposed children. Child Neuropsychology. 2018 Jul 4;24(5):686-701.

^8^ Boivin MJ, Zoumenou R, Sikorskii A, Fievet N, Alao J, Davidson L, Cot M, Massougbodji A, Bodeau-Livinec F. Neurodevelopmental assessment at one year of age predicts neuropsychological performance at six years in a cohort of West African Children. Child Neuropsychology. 2021 May 19;27(4):548-71.

^9^ Garrison A, Boivin MJ, Fiévet N, Zoumenou R, Alao JM, Massougbodji A, Cot M, Bodeau-Livinec F. The Effects of Malaria in Pregnancy on Neurocognitive Development in Children at 1 and 6 Years of Age in Benin: A Prospective Mother–Child Cohort. Clinical Infectious Diseases. 2021 Jul 23.

^10^ Mireku MO, Cot M, Massougbodji A, Bodeau-Livinec F. Relationship between stunting, wasting, underweight and geophagy and cognitive function of children. Journal of tropical pediatrics. 2020 Oct;66(5):517-27.

^11^ Mireku MO, Boivin MJ, Davidson LL, Ouédraogo S, Koura GK, Alao MJ, Massougbodji A, Cot M, Bodeau-Livinec F. Impact of helminth infection during pregnancy on cognitive and motor functions of one-year-old children. PLoS neglected tropical diseases. 2015 Mar 10;9(3):e0003463.

^12^ Bodeau-Livinec F, Davidson LL, Zoumenou R, Massougbodji A, Cot M, Boivin MJ. Neurocognitive testing in West African children 3–6 years of age: Challenges and implications for data analyses. Brain research bulletin. 2019 Feb 1;145:129-35.

^13^ Koura GK, Boivin MJ, Davidson LL, Ouédraogo S, Zoumenou R, Alao MJ, Garcia A, Massougbodji A, Cot M, Bodeau-Livinec F. Usefulness of child development assessments for low-resource settings in francophone Africa. Journal of developmental and behavioral pediatrics: JDBP. 2013 Sep;34(7).

^14^ Boivin MJ, Nakasujja N, Familiar I, Murray SM, Sikorskii A, Awadu J, Shohet C, Givon D, Ruiseñor-Escudero H, Schut EE, Opoka RO. Effect of caregiver training on neurodevelopment of HIV-exposed uninfected children and caregiver mental health: a Ugandan cluster randomized controlled trial. Journal of developmental and behavioral pediatrics: JDBP. 2017 Nov;38(9):753.

^15^ Boivin MJ, Bangirana P, Nakasujja N, Page CF, Shohet C, Givon D, Bass JK, Opoka RO, Klein PS. A year-long caregiver training program to improve neurocognition in preschool Ugandan HIV-exposed children. Journal of developmental and behavioral pediatrics: JDBP. 2013 May;34(4):269.

^16^ Boivin MJ, Maliwichi-Senganimalunje L, Ogwang LW, Kawalazira R, Sikorskii A, Familiar-Lopez I, Kuteesa A, Nyakato M, Mutebe A, Namukooli JL, Mallewa M. Neurodevelopmental effects of ante-partum and post-partum antiretroviral exposure in HIV-exposed and uninfected children versus HIV-unexposed and uninfected children in Uganda and Malawi: a prospective cohort study. The lancet HIV. 2019 Aug 1;6(8):e518-30.

^17^ Familiar I, Nakasujja N, Bass J, Sikorskii A, Murray SM, Ruisenor-Escudero H, Bangirana P, Opoka R, Boivin MJ. Caregivers' depressive symptoms and parent-report of child executive function among young children in Uganda. Learning and individual differences. 2016 Feb 1;46:17-24.

**Supplementary Table 2. Cognitive development scores of children born to adult mothers (pooled data from sub-Saharan Africa) compared to the Mullen Scales of Early Learning reference group**

| **Child cognitive development (Mullen scales of Early Learning; T-Scores)** | ***Mean (SD)*** | | ***t, p*-value** |
| --- | --- | --- | --- |
|  | **Pooled data from sub-Saharan Africa (n=2481)** | **Mullen Scales reference group (USA population)** |  |
| Composite score of early learning^a^ | 93.4 (14.6) | 100 (20) | **-13.28, <0.0001** |
| Gross motor | 45.7 (10.6) | 50 (10) | **-14.70, <0.0001** |
| Visual reception | 46.7 (10.7) | 50 (10) | **-11.22, <0.0001** |
| Fine motor | 47.0 (9.7) | 50 (10) | **-10.73, <0.0001** |
| Receptive language | 44.8 (7.7) | 50 (10) | **-20.52, <0.0001** |
| Expressive language | 46.4 (10.3) | 50 (10) | **-12.49, <0.0001** |

Gross motor scores n=848 |^a^Early Learning Composite Score: Age standardised composite score of fine motor, visual reception, expressive language, receptive language domains (range 49-155)

**Supplementary Table 3. Cognitive development scores of children born to adolescent mothers compared to children born to adult mothers (pooled data from sub-Saharan Africa)**

| **Child cognitive development (Mullen scales of Early Learning; T-Scores)** | ***Mean (SD)*** | | ***t, p*-value** |
| --- | --- | --- | --- |
|  | **Children born to adolescent mothers (n=954)** | **Pooled estimated from sub-Saharan Africa (n=2481)** |  |
| Child age (months) | 18.6 (14.7) | 17.3 (2.8) | **4.21, <0.0001** |
| Composite score of early learning^a^ | 93.5 (21.3) | 93.4 (14.6) | 0.16, 0.87 |
| Gross motor | 49.8 (12.5) | 45.7 (10.6) | **9.64, <0.0001** |
| Visual reception | 42.2 (14.0) | 46.7 (10.7) | **-10.09, <0.0001** |
| Fine motor | 43.9 (14.7) | 47.0 (9.7) | **-7.19, <0.0001** |
| Receptive language | 47.6 (13.5) | 44.8 (7.7) | **7.60, <0.0001** |
| Expressive language | 51.7 (13.3) | 46.4 (10.3) | **12.41, <0.0001** |

Gross motor scores n=848 |^a^Early Learning Composite Score: Age standardised composite score of fine motor, visual reception, expressive language, receptive language domains (range 49-155)

**Supplementary Table 4. Cognitive development scores of children born to adolescent mothers living with HIV compared to children born to adult mothers (pooled data from sub-Saharan Africa) classified as HIV exposed uninfected**

| **Child cognitive development (Mullen scales of Early Learning; T-Scores)** | ***Mean (SD)*** | | ***t, p*-value** |
| --- | --- | --- | --- |
|  | **Children born to adolescent mothers (n=230)** | **Pooled estimated from sub-Saharan Africa (n=1097)** |  |
| Child age (months) | 24.4 (16.9) | 22.0 (5.5) | **3.84, 0.0001** |
| Composite score of early learning^a^ | 91.7 (22.7) | 89.4 (15.2) | 1.89, 0.06 |
| Gross motor | 47.3 (13.4) | 40.6 (8.0) | **10.08, <0.0001** |
| Visual reception | 41.1 (13.9) | 43.3 (11.2) | **-2.59, 0.01** |
| Fine motor | 42.0 (15.5) | 38.9 (10.3) | **3.75, 0.0002** |
| Receptive language | 46.8 (14.7) | 39.2 (8.5) | **10.63, <0.0001** |
| Expressive language | 51.3 (14.3) | 39.5 (10.1) | **14.87, <0.0001** |

Gross motor scores n=848 |^a^Early Learning Composite Score: Age standardised composite score of fine motor, visual reception, expressive language, receptive language domains (range 49-155)

**Supplementary Table 5. Cognitive development scores of children born to adolescent mothers not living with HIV compared to children born to adult mothers (pooled data from sub-Saharan Africa) classified as developing as expected**

| **Child cognitive development (Mullen scales of Early Learning; T-Scores)** | ***Mean (SD)*** | | ***t, p*-value** |
| --- | --- | --- | --- |
|  | **Children born to adolescent mothers (n=724)** | **Pooled estimated from sub-Saharan Africa (n=1384)** |  |
| Child age (months) | 16.8 (13.4) | 13.6 (0.7) | **8.86, <0.0001** |
| Composite score of early learning^a^ | 94.0 (20.7) | 96.6 (14.2) | **-3.39, 0.0007** |
| Gross motor | 50.4 (12.2) | 49.7 (12.7) | 1.21, 0.22 |
| Visual reception | 42.5 (14.3) | 49.3 (10.2) | **-12.60, <0.0001** |
| Fine motor | 44.5 (14.3) | 49.7 (10.0) | **-9.73, <0.0001** |
| Receptive language | 47.8 (13.1) | 45.8 (7.7) | **4.41, <0.0001** |
| Expressive language | 51.8 (13.0) | 47.3 (10.3) | **8.68, <0.0001** |

Gross motor scores n=848 |^a^Early Learning Composite Score: Age standardised composite score of fine motor, visual reception, expressive language, receptive language domains (range 49-155)


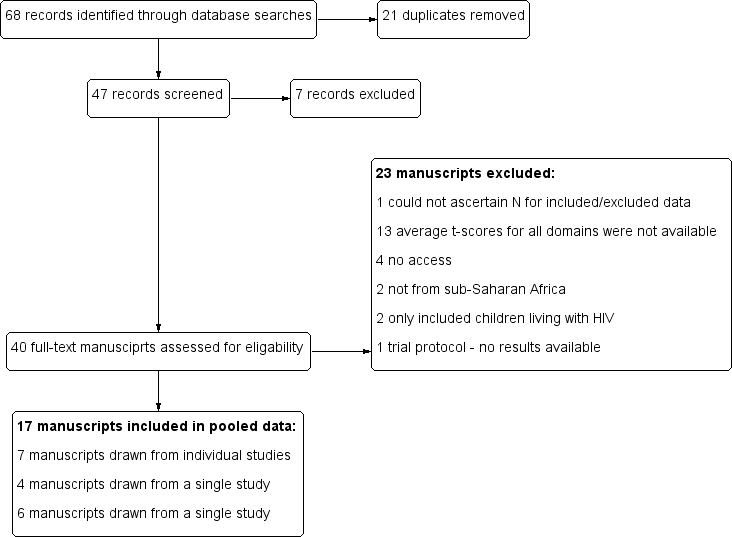


**Supplementary Figure 1: Flow diagram of sub-Saharan African studies included in pooled data *(t-scores)* for Mullen Scales of Early Learning**
